# Toward on-site food authentication using nanopore sequencing

**DOI:** 10.1016/j.fochx.2019.100035

**Published:** 2019-06-05

**Authors:** Marleen M. Voorhuijzen-Harink, Rico Hagelaar, Jeroen P. van Dijk, Theo W. Prins, Esther J. Kok, Martijn Staats

**Affiliations:** a Wageningen University & Research – Wageningen Food Safety Research (WFSR), Netherlands; b Princess Máxima Center for Pediatric Oncology, Netherlands

## Abstract

• MinION DNA metabarcoding is a promising tool for species identification in food.
• MinION and Illumina MiSeq sequencing platforms perform equally accurate.
• Species identification with MinION sequencing requires dedicated bioinformatics.

## 1 Introduction

Food fraud is a major concern as it has economic impact and affects consumer confidence (Grunert, 2002, Spink and Moyer, 2011). Deliberate adulteration of a product for financial profit may have far-reaching consequences with for the food industry, as observed with the horsemeat scandal in 2013 (O’Mahony, 2013). This scandal raised questions about the effectiveness of controls along the food chain by businesses and governmental authorities. In case of a food safety risk or indications of fraudulent raw materials food inspection services are required to act fast. Incidents like the horsemeat scandal call for methods that allow the rapid assessment of the authenticity and quality of raw materials and food commodities.

A promising fast detection technology is the MinION DNA sequencer from Oxford Nanopore Technologies (ONT) which is smaller than a smartphone and able to produce data within minutes. The sequencer with low purchasing price is able to sequence individual DNA molecules as they drive through biological nanopores by an applied electrical field (Loman & Watson, 2015). Currently, MinION sequencing shows a higher error rate compared to traditional Next-Generations Sequencing (NGS) equipment such as Illumina MiSeq technology (Judge et al., 2015, Laver et al., 2015). Nonetheless, the technology is already used for fast medical applications, even in the field, for instance for the mobile real-time surveillance of Zika virus in Brazil (Faria et al., 2016). Recently, Menegon et al. (2017) showed that DNA barcoding and MinION sequencing can also be used in on-site biodiversity assessments, despite the reported limitations of the technology.

So far, no food-related applications of the MinION technology have been reported. The technology has the potential to screen complex food products quickly and comprehensively for a multitude of DNA-based markers. Here, we assessed the potential of MinION-based DNA metabarcoding as a system that may lead to fast food authentication, without the need for a highly priced sequencing platform. Two experimental fish mixtures were prepared and metabarcoded, after which the performance, i.e. the number and level of correctly identified fish species, of MinION sequencing was compared to the Illumina MiSeq technology.

Fish was selected as example of food products vulnerable to food fraud (Everstine et al., 2013, Ogden, 2008, Warner et al., 2013). The mitochondrial DNA barcodes cytochrome oxidase subunit I gene (COI) (Ward, Hanner, & Hebert, 2009) and cytochrome *b* (*cyt*b) gene (Griffiths et al., 2014) were selected as they have proven to be effective in identifying fish products (Cawthorn et al., 2012, Hanner et al., 2011, Logan et al., 2008, Miller and Mariani, 2010, Wong and Hanner, 2008).

In this paper, the applicability of the MinION device for effective metabarcoding has been assessed. Furthermore, in order to process the typical quality of MinION reads a dedicated bioinformatics workflow was developed and the degree of run-to-run contamination was determined.

## 2 Material and methods

### 2.1 Species materials and DNA extraction

Fish species were collected during fishing surveys in the North Sea and the Atlantic Ocean, and taxonomically characterised by Wageningen Marine research in September 2013 or obtained from the Dutch Food and Consumer Product Safety Authority (NVWA) – Ministry of Economic Affairs and Climate Policy. All specimens were stored at −20 °C.

Two fish mixtures were prepared by chopping partially frozen fish specimens and mixing on fresh weight basis. Muscle tissue was used, except for *Zoarces viviparus, Scophthalmus rhombus*, *Myoxocephalus scorpius* and *Agonus cataphractus*, which were partially or completely used to reach the required amount of fresh weight (Table 2a, Table 2b). Mixtures were lyophilized, ground into powder, mixed homogenously by tumbling in a head-over-head tumbler and stored at −20 °C.

DNA was isolated from mixtures or individual species, according to a modified cetyl-trimethyl-ammonium-bromide (CTAB) procedure (Arulandhu et al., 2017). The DNA pellet was suspended in TE, and stored at 4 °C. Quality and quantity of the DNA was assessed using the NanoDrop spectrophotometer (NanoDrop ND-1000, NanoDrop Technologies, DE, USA).

### 2.2 DNA barcoding of reference materials

The COI-3 primer cocktail of Ivanova, Zemlak, Hanner, and Hebert (2007) and the FishcytB-F and CytB1-5R primers of Sevilla et al. (2007) were used for DNA barcoding of reference materials. COI-3 PCR reactions were performed in a reaction volume of 25 μl containing 50 ng of genomic DNA, 1X HotStarTaq Master Mix (Qiagen), 0.2 μM of forward and 0.2 μM reverse primer cocktail. Amplification conditions were: 95 °C for 900 s, 35 cycles of 94 °C for 30 s, 52 °C for 40 s, and 72 °C for 60 s, and a final extension step of 72 °C for 600 s. *Cyt*b PCR reactions were performed in a reaction volume of 25 μl containing 50 ng of genomic DNA, 1X HotStarTaq Master Mix (Qiagen), and 0.1 μM of each primer. Amplification conditions were: 95 °C for 900 s, 35 cycles of 94 °C for 30 s, 50 °C for 40 s, and 72 °C for 60 s, and a final extension step of 72 °C for 600 s.

PCR products were purified using the QIAquick PCR purification kit according to the manufacturer’s protocol (Qiagen) prior to Sanger sequencing (Macrogen Inc.). Species identity of the single specimens was confirmed by querying COI and *cyt*b Sanger sequences using nucleotide BLAST (BLASTn, NCBI).

### 2.3 DNA metabarcoding and Oxford Nanopore Technologies (ONT) MinION sequencing

The COI-3 and *cyt*b primers as described above but without M13 tails were used for DNA metabarcoding the fish mixtures. PCR products of three independent PCR reactions of the same DNA extract were mixed prior to purification using the QIAquick PCR purification kit. MinION libraries were prepared out of 1 μg purified PCR product using the SQK-LSK108 Nanopore Sequencing Kit (ONT) according to the manufacturer’s protocol. Flow cells, versions R9.4/FLO-MIN106 and R9.5/FLO-MIN107, were quality-checked upon arrival using the MinKNOW software (version 1.7.3) to ensure the presence of at least 800 active biological nanopores per device. Flow cells were used twice, with washing steps in between using the Flow Cell Wash Kit (provided by ONT). Barcode marker *cyt*b was analysed in the first and COI in the second run of the flow cells. Experimental fish mixture B was independently analysed twice to verify the repeatability of the procedure. The run time of the MinION flow cells varied from 12 to 35 min, and between 39,000 and 93,000 reads were generated per run. The extent of run-to-run contamination was determined by counting the number of *cyt*b reads in the COI sequencing run experiment.

### 2.4 ONT data analysis workflow

Basecalling of the FAST5 files was performed using albacore version 2.0.2, keeping only FASTQ reads that passed the PASS classification. FASTQ reads with an average Phred score of at least 10, and read lengths between 500 and 800 bases were selected using PRINSEQ 0.20.4 (Schmieder & Edwards, 2011). Porechop (v0.2.1: https://github.com/rrwick/Porechop) was used to remove adapters with default settings. Reads containing the *cyt*b or COI DNA barcode primers were selected with 20% error rate and a minimum overlap of 15 nt using Cutadapt 1.9.1 (Martin, 2011). Porechop was used to trim off the *cyt*b or COI DNA barcode primers.

Clustering was performed using cdhit-est 4.6 with 80% identity (Huang, Niu, Gao, Fu, & Limin, 2010). Multiple sequence alignment (MSA) was performed using MAFFT v7.266 with – adjust direction enabled and with the L-INS-I iterative refinement method selected (Katoh & Standley, 2013). For clusters with more than 100 reads, 100 reads were randomly selected prior to MSA. Consensus sequences were extracted from the MSAs using a 30% majority consensus procedure i.e. nucleotide positions in the alignment with the highest percent-identity, and with a percent-identity higher than the 30% threshold were selected. All DNA barcode primer residues that remained after consensus building were removed with Porechop.

To assign consensus sequences to taxonomy, standalone BLASTn megablast searches (BLASTN v2.2.31+; Altschul, 1997) were performed against the nt/nr database (accessed July 2017). Consensus sequences were assigned to the database sequence to which they align, based on bit score, having at least 98% sequence identity and a minimum of 90% query coverage. The BLAST output was interpreted following the guideline of Arulandhu et al. (2017).

NGS datasets were deposited in the European Nucleotide Archive (ENA) under accession study number PRJEB23856.

### 2.5 Error estimation of nanopore reads

Nanopore reads from cdhit-est clusters larger than 100 were aligned against their respective consensus sequences using a nucleotide substitution penalty of 1 (-q1), gap opening penalty of 1 (option –a1) and a gap extension penalty of 1 (option –b1) using the LAST (Frith, Hamada, & Paul, 2010) aligner (version 914). LAST alignments were converted to bam using SAMtools (v1.6; Li et al., 2009). The error counts (mismatches, insertions, deletions) were calculated from SAM alignment files using the script provided in Quick, Quinlan, and Loman (2014). The percentage of errors was defined as 100 × (sum of mismatches + deletions + insertions)/(sum of read lengths).

### 2.6 Illumina MiSeq NGS procedure

COI and *cyt*b PCR amplicons were prepared for the two fish mixtures following the same procedure as described for DNA metabarcoding and MinION sequencing. Prior to NGS, COI or *cyt*b amplicons were extended with Illumina flow cell adapter sequences and a sample-specific barcode sequence using fusion primers custom designed by the Wageningen Bioveterinary Research (WBVR, part of Wageningen University and Research, Lelystad, the Netherlands). COI and *cyt*b amplicons of each sample were given the same multiplex identifier (MID). The quality of the amplicon libraries was evaluated using a Bioanalyzer with a High Sensitivity DNA Kit (Agilent Technologies) and the quantities were determined using a Quibit dsDNA HS Assay Kit (Life Technologies). Cluster generation and paired-end 300-bp sequencing was done on an Illumina MiSeq V2 instrument at WBVR. Raw Illumina reads were sorted per sample using the MIDs. The “CITESspeciesDetect” workflow of Arulandhu et al. (2017) was applied on the Illumina MiSeq data, but with using regular (un-anchored) adapter trimming and with the overlap parameter set at 20 using Cutadapt (Martin, 2011).

## 3 Results & discussion

In this study, the combination of DNA metabarcoding and MinION sequencing was tested for the identification of fish species in two well-characterised experimental mixtures, containing six and eleven species (Table 1, Table 2a). Additionally, a dedicated bioinformatics workflow was developed taking into account the characteristics of MinION reads. The MinION-based DNA metabarcoding strategy resulted in the reliable identification of all species within the prepared fish mixtures.

**Table 1:** Composition of fish mixture A and level of identification resulting from MinION and Illumina MiSeq sequencing.

Ingredient	Weight fraction (%)	MinION Flow cell chemistry R9.4			Illumina MiSeq		
		*cyt*b	COI	Final conclusion	*cyt*b	COI	Final conclusion
*Pleuronectes platessa*	75	*P. platessa*	*P. platessa*	*P. platessa*	*P. platessa*	*P. platessa*	*P. platessa*
*Limanda limanda*	5	*L. limanda*	*L. limanda*	*L. limanda*	*L. limanda*	*L. limanda*	*L. limanda*
*Microstomus kitt*	5	*M. kitt*	*M. kitt*	*M. kitt*	*M. kitt*	*M. kitt*	*M. kitt*
*Limanda aspera*	5	*	*L. aspera*	*L. aspera*	*	*L. aspera*	*L. aspera*
*Platichthys flesus*	5	*P. flesus*	*P. flesus*	*P. flesus*	*P. flesus*	*P. flesus*	*P. flesus*
*Scophthalmus rhombus*	5	*S. rhombus*	‡	*S. rhombus*	*S. rhombus*	‡	*S. rhombus*

* No sequencing reads.

‡ No PCR barcode amplification.

**Table 2a:** Composition of fish mixture B and level of identification resulting from MinION sequencing, with two flow cell chemistries.

Ingredient	Weight fraction (%)	MinION Flow cell chemistry R9.4			MinION Flow cell chemistry R9.5		
		*cyt*b	COI	Final conclusion	*cyt*b	COI	Final conclusion
*Microstomus kitt*	17	*M. kitt*	*M. kitt*	*M. kitt*	*M. kitt*	*M. kitt*	*M. kitt*
*Pleuronectes platessa*	8	*P. platessa*	*P. platessa*	*P. platessa*	*P. platessa*	*P. platessa*	*P. platessa*
*Limanda limanda*	8	*L. limanda*	*Limanda*	*L. limanda*	*L. limanda*	*L. limanda*	*L. limanda*
*Limanda aspera*	8	*	*L. aspera*	*L. aspera*	*L. aspera*	*L. aspera*	*L. aspera*
*Platichthys flesus*	8	*P. flesus*	*P. flesus*	*P. flesus*	*P. flesus*	*P. flesus*	*P. flesus*
*Agonus cataphractus*	8	*Agonidae*	*A. cataphractus*	*A. cataphractus*	*Agonidae*	*A. cataphractus*	*A. cataphractus*
*Myoxocephalus scorpius*	8	*M. scorpius*	*Myoxocephalus*	*M. scorpius*	*M. scorpius*	*Myoxocephalus*	*M. scorpius*
*Hippoglossoides platessoides*	8	*H. platessoides*	*H. platessoides*	*H. platessoides*	*H. platessoides*	*H. platessoides*	*H. platessoides*
*Zoarces viviparus*	8	*Z. viviparus*	*Z. viviparus*	*Z. viviparus*	*Z. viviparus*	*Z. viviparus*	*Z. viviparus*
*Osmerus eperlanus*	8	‡	*O. eperlanus*	*O. eperlanus*	‡	*O. eperlanus*	*O. eperlanus*
*Scophthalmus maximus*	8	*S. maximus*	‡	*S. maximus*	*S. maximus*	‡	*S. maximus*

* No sequencing reads.

‡ No PCR barcode amplification.

The molecular identification was based on full-length DNA barcodes cytb and COI that have been widely used for the identification of a large number of commercially important fish species (Ivanova et al., 2007, Sevilla et al., 2007). Individual species were taxonomically determined and separately barcoded with Sanger sequencing. All sequences confirmed the initial taxonomic assessment (Table 1, Table 2a), though for three of the 12 species only one of the barcodes resulted in an amplicon underlining the necessity of using the combination of the two barcode markers for fish species identification. Next, metabarcoding was applied to the two mixtures, using the same barcodes, followed by MinION sequencing.

**Fig. 1:**
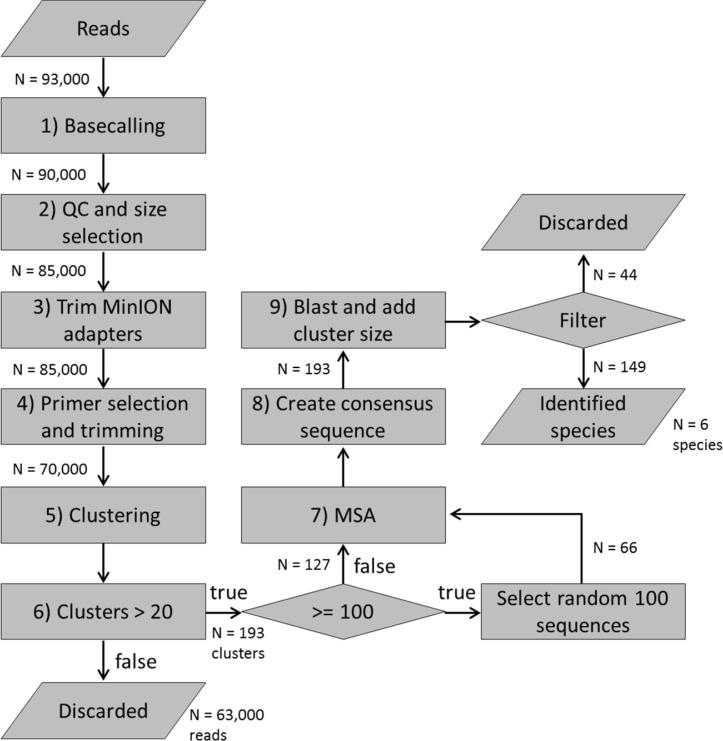
Schematic representation of the bioinformatics workflow for identifying species using MinION sequencing data. Indicated are also the results of mixture A, *cyt*b: read counts until step 5, cluster counts from step 6 onwards, and number of identified species in the final step. Similar numbers were observed for mixture A, COI and the runs of mixture B.

Accurate species identification within mixtures using DNA metabarcoding is highly dependent on the discriminative power of the applied bioinformatics workflow. The sequence similarity between amplicons of different species generated with the same barcode marker can be very high (Ward et al., 2009), making correct identification down to species level challenging. When using ONT’s MinION system as NGS platform, correct identification is even more challenging as a result of the error rate of individual MinION reads, which was here estimated to be ∼13% (not shown). Since the use of such error-prone individual reads is not suitable for accurate species identification, an effective DNA metabarcoding workflow was developed using common bioinformatics tools for generating error-corrected consensus DNA barcodes. The workflow (Fig. 1) consisted of steps for preprocessing of reads (steps 1–4) followed by clustering of reads using CD-HIT-est (Li and Godzik, 2006, Fu et al., 2012; step 5). CD-HIT uses a fast greedy algorithm that is normally used to identify representative sequences for each cluster. Here, the reads assigned to each cluster were passed to MAFFT (Katoh & Standley, 2013) for multiple sequence alignment (MSA) and consensus calling (step 6–8). This procedure yielded multiple error-corrected consensus reads per DNA barcode marker suitable for accurate species identification with BLAST (step 9). A 98% BLAST sequence identity threshold was used, which is commonly used to delimit commercial fish species (Handy, 2011). A bioinformatics workflow for processing MinION data with a similar MAFFT-based sequence consensus calling step, but without prior clustering of reads was published for DNA barcoding of specimens of insects (Srivathsan et al., 2018). In our workflow, CD-HIT clustering of MinION reads prior to MAFFT-based sequence consensus calling is necessary to group sequences that are related and derived from individual species in a mixture, and thus to enable multi-species identification in complex mixtures. By subsampling clusters with more than 100 reads, it was found that the total analysis time could be restricted to 5–6 h using a 6-core (Intel Xeon) workstation. Also, using over 100 reads per cluster did not result in better identification. Basecalling was particularly computationally intense, providing a potential bottleneck for fast identification.

The dedicated DNA metabarcoding workflow for processing MinION reads was applied to the COI and *cyt*b data generated for the two fish mixtures. Correct identification of all species was achieved without any false discoveries using the combined COI and *cyt*b data (Table 1, Table 2a). For mixture A, correct species identification was achieved with both barcodes in four out of the six species, and single barcode species identification for the other two. For *Limanda aspera*, *cyt*b did not lead to MinION reads with quality meeting the set criteria and for *Scophthalmus rhombus* no PCR amplicon was observed with COI. The inability to amplify COI from *S. rhombus* was determined beforehand during DNA barcoding of the reference materials. For mixture B two replicate experiments were performed with two MinION flow cell chemistries (Table 2a). Using flow cell chemistry R9.4, double barcode identification was achieved for five out of the eleven species, and single barcode identification for the other six. Similar to the single barcode identifications in mixture A, *Scophthalmus maximus* and *Osmerus eperlanus* did not show PCR amplicons for COI or *cyt*b, respectively, and *L. aspera* did not lead to MinION reads for *cyt*b. Furthermore, species level identification was seen for *Limanda limanda* and *Myoxocephalus scorpius* with *cyt*b and genus level with COI. In case of *M. scorpius* the genus level identification with COI is a result of the lack of genetic variability. *Agonus cataphractus* was identified with COI at species level and with *cyt*b at family level as a result of database incompleteness for *cyt*b for this fish species. Mixture B and flow cell chemistry R9.5 resulted in slightly improved results: *L. limanda* and *L. aspera* were identified with both DNA barcode markers at species level. *M. scorpius* and *A. cataphractus* were again identified at species level with a single barcode (*cyt*b and COI respectively), the other barcode provided genus or family level resolution, again due to the lack of genetic variability or database incompleteness. In all cases, the identifications at the genus- and family level did not contradict the species results but emphasize the importance of using two barcode markers for fish species identification. Additionally, the results showed the impact of the developed bioinformatics workflow: application enabled the reduction in redundancy of almost 100 K of raw reads into 193 consensus reads (Fig. 1), which consequently resulted in a reduction of the error rate from ∼13% for the individual reads to less than 2% for the consensus reads, or in other words over 98% barcode identity for all identified species.

MinION flow cells can be re-used although, as indicated in the corresponding manual, applying ONT’s Flow Cell Wash Kit (EXP-WSH002) will remove only most of the initial library. Molecules of the previous run will likely be present and sequenced in the next run. For species identification, this may lead to false conclusions on sample composition. The here applied strategy addressed this problem by the primer selection step in the bioinformatics workflow and simultaneously enabled the assessment of the level of surplus reads in the second run since different barcode markers in consecutive runs were used. Approximately 14% of the total amount of reads in the COI sequencing runs were identified as *cyt*b reads, thereby underpinning the necessity of using different barcode markers in consecutive runs or sample tagging.

The performance of MinION-based DNA metabarcoding was compared with the results of Illumina MiSeq sequencing using the same fish mixtures and DNA barcode markers (Table 1, Table 2b). Both techniques obtained near-identical results, meaning that using the Illumina MiSeq technology all species in both mixtures could also unequivocally be identified (Table 2b). Small differences between the two sequencing platforms were observed in the data for mixture B, regarding the COI identification of *Zoarces viviparous*: the MinION system resulted in identification down to species level while family-level resolution was provided using the MiSeq system. Here the chemistry of the MiSeq run yielded maximum sequence lengths of 300 bp, while with the MinION system, reads typically covered the entire barcode amplicons. With both systems identification down to species level for this individual species was reached using the *cyt*b barcode marker (Table 2a, Table 2b), resulting in the same final identification. The results show that for these mixtures and barcodes both NGS platforms perform equally well.

**Table 2b:** Composition of fish mixture B and level of identification resulting from Illumina MiSeq sequencing.

Ingredient	Weight fraction (%)	Illumina MiSeq		
		*cyt*b	COI	Final conclusion
*Microstomus kitt*	17	*M. kitt*	*M. kitt*	*M. kitt*
*Pleuronectes platessa*	8	*P. platessa*	*P. platessa*	*P. platessa*
*Limanda limanda*	8	*L. limanda*	*L. limanda*	*L. limanda*
*Limanda aspera*	8	*	*L. aspera*	*L. aspera*
*Platichthys flesus*	8	*P. flesus*	*P. flesus*	*P. flesus*
*Agonus cataphractus*	8	*Agonidae*	*A. cataphractus*	*A. cataphractus*
*Myoxocephalus scorpius*	8	*M. scorpius*	*Myoxocephalus*	*M. scorpius*
*Hippoglossoides platessoides*	8	*H. platessoides*	*H. platessoides*	*H. platessoides*
*Zoarces viviparus*	8	*Z. viviparus*	*Zoarcidae*	*Z. viviparus*
*Osmerus eperlanus*	8	‡	*O. eperlanus*	*O. eperlanus*
*Scophthalmus maximus*	8	*S. maximus*	‡	*S. maximus*

* No sequencing reads.

‡ No PCR barcode amplification.

## 4 Conclusions

Here, MinION-based DNA metabarcoding was assessed for identification of fish species in complex mixtures. A dedicated bioinformatics workflow was developed using freely available tools that enables processing of MinION reads to generate multiple high-quality consensus DNA barcodes suitable for multi-species identification. The MinION sequencing platform proved for this strategy to be highly effective and at least as accurate as the Illumina MiSeq platform. The high accuracy and practicability of the MinION sequencer make MinION-based DNA metabarcoding promising for fast analysis of complex food and feed mixtures. Furthermore, because of the low purchasing price of the MinION sequencer, standard laboratories can perform sequencing analyses in-house, thereby decreasing the sample throughput time significantly compared to traditional NGS sequencing technologies.

## Declaration of Competing Interest

The authors declare that they have no known competing financial interests.
